# Differential Pregnancy Decisions in a Woman With a Recurrent Prenatal Diagnosis of Hypophosphatasia

**DOI:** 10.7759/cureus.87005

**Published:** 2025-06-29

**Authors:** Taiyo Oguro, Ryuhei Nagai, Yuta Shimomoto

**Affiliations:** 1 Obstetrics and Gynecology, Kochi Medical School, Kochi, JPN

**Keywords:** decision-making process, genetic counseling, hypophosphatasia, prenatal diagnosis, rare disease

## Abstract

Hypophosphatasia (HPP) is a rare inherited bone disorder caused by ALPL gene mutations, leading to reduced alkaline phosphatase (ALP) activity and impaired bone mineralization. Although its prevalence is relatively higher in Japan, prenatal diagnosis remains challenging. In particular, obstetricians and clinical geneticists, who are typically involved in prenatal assessment, often have limited experience with this condition due to its rarity. Among the various types of HPP, the prenatal benign form has a favorable prognosis, yet awareness and understanding of this specific subtype are limited.

We report a case involving two separate pregnancies in the same woman, both diagnosed prenatally with HPP. During the second pregnancy, counseling was provided at 21 weeks by a physician. Given the rarity of HPP, even specialists such as obstetricians, pediatricians, or clinical geneticists often have limited direct clinical experience with the condition. Consequently, the counseling leaned toward a negative outlook, and the patient opted for termination. In contrast, during the third pregnancy, counseling began at nine weeks with detailed and balanced information, informed by the previous case and supported by a multidisciplinary team. This time, the patient elected to continue the pregnancy. The neonate was diagnosed with prenatal benign HPP and showed favorable clinical progress.

This case highlights how the timing, quality, and balance of prenatal counseling can significantly influence parental decision-making in pregnancies complicated by rare diseases. In cases of rare conditions such as HPP, early and accurate counseling by an experienced multidisciplinary team is essential to support informed and autonomous decisions.

## Introduction

Hypophosphatasia (HPP) is a rare inherited disorder of bone mineralization caused by mutations in the ALPL gene, which encodes the tissue-nonspecific alkaline phosphatase (TNSALP). This results in decreased ALP activity and the subsequent disruption of skeletal development [1,2]. HPP is classified into six clinical forms based on the age of onset and clinical presentation: perinatal severe, prenatal benign, infantile, childhood, adult, and odontohypophosphatasia. Younger patients with these variants have been included in expanded clinical nosology for pediatric HPP, based on long-term data [3]. HPP is a rare inherited bone disorder with a global incidence estimated between one in 100,000 and one in 300,000 births. Japan is no exception, with the incidence of severe HPP estimated at around one in 150,000 births [4]. Both the perinatal benign and lethal forms of HPP are classified under the same OMIM entry number (#241500), reflecting their phenotypic spectrum.

Two specific ALPL mutations, p.Leu520ArgfsX86 and p.Phe327Leu, are common in Japanese individuals. The p.Phe327Leu mutation is particularly associated with the prenatal benign form, which generally has a good prognosis [5]. This mutation and others, such as p.Phe310Leu and c.1559delT, have been associated with distinct phenotypes in Japanese patients with HPP [6]. Despite this, prenatal diagnosis remains difficult, and only a limited number of physicians are familiar with the condition. Moreover, the centralization of perinatal medical services in Japan is still insufficient, making it even more challenging for providers to access expert consultation. As a result, many clinicians have little practical experience with HPP, and awareness of its prognosis remains low.

Historically, treatment for HPP has been symptomatic, focusing on managing respiratory insufficiency, seizures, and hypercalcemia in severe cases. However, the recent development of enzyme replacement therapy using asfotase alfa has significantly improved outcomes. The five-year survival rate of patients receiving asfotase alfa has been reported as 84%, compared to 27% without treatment [7].

Although prenatal benign HPP generally has a favorable prognosis, it is not well known among general obstetricians and pediatricians. This lack of familiarity can lead to inadequate counseling and may influence critical decision-making during pregnancy. Here, we present a case involving two pregnancies with a prenatal diagnosis of HPP in the same patient, where different decisions were made based on the timing and quality of prenatal counseling.

## Case presentation

A 33-year-old woman was referred to our facility during her second and third pregnancies, both of which involved fetuses diagnosed prenatally with HPP. Her first pregnancy had been uneventful, with no complications during the prenatal course and no abnormalities in the newborn; however, due to arrest of labor, delivery was performed by cesarean section.

**Figure 1:**
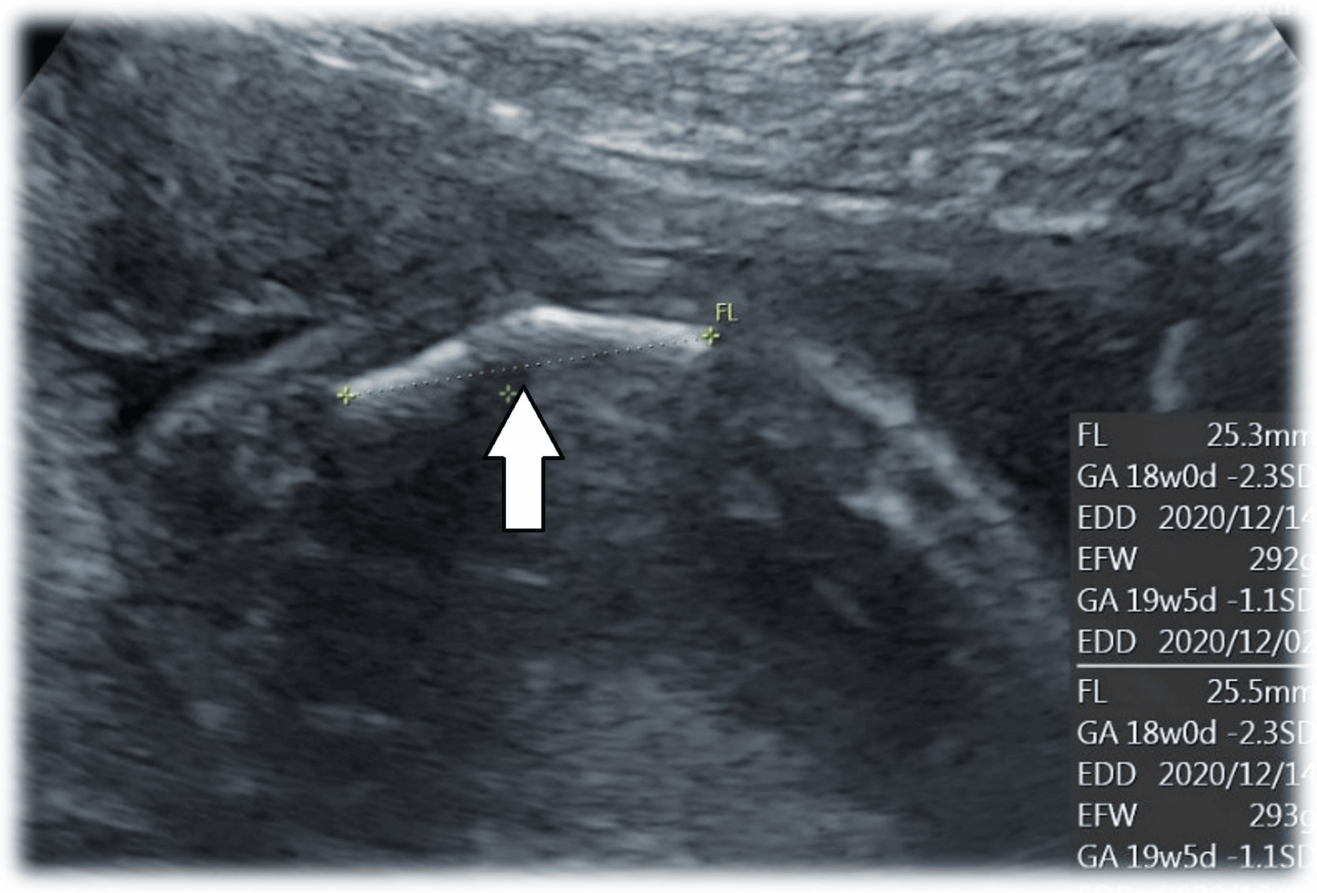
Femur length on ultrasonography of the second pregnancy at 20 weeks and four days of gestation The fetal femur was shortened and curved (white arrow).

**Figure 2:**
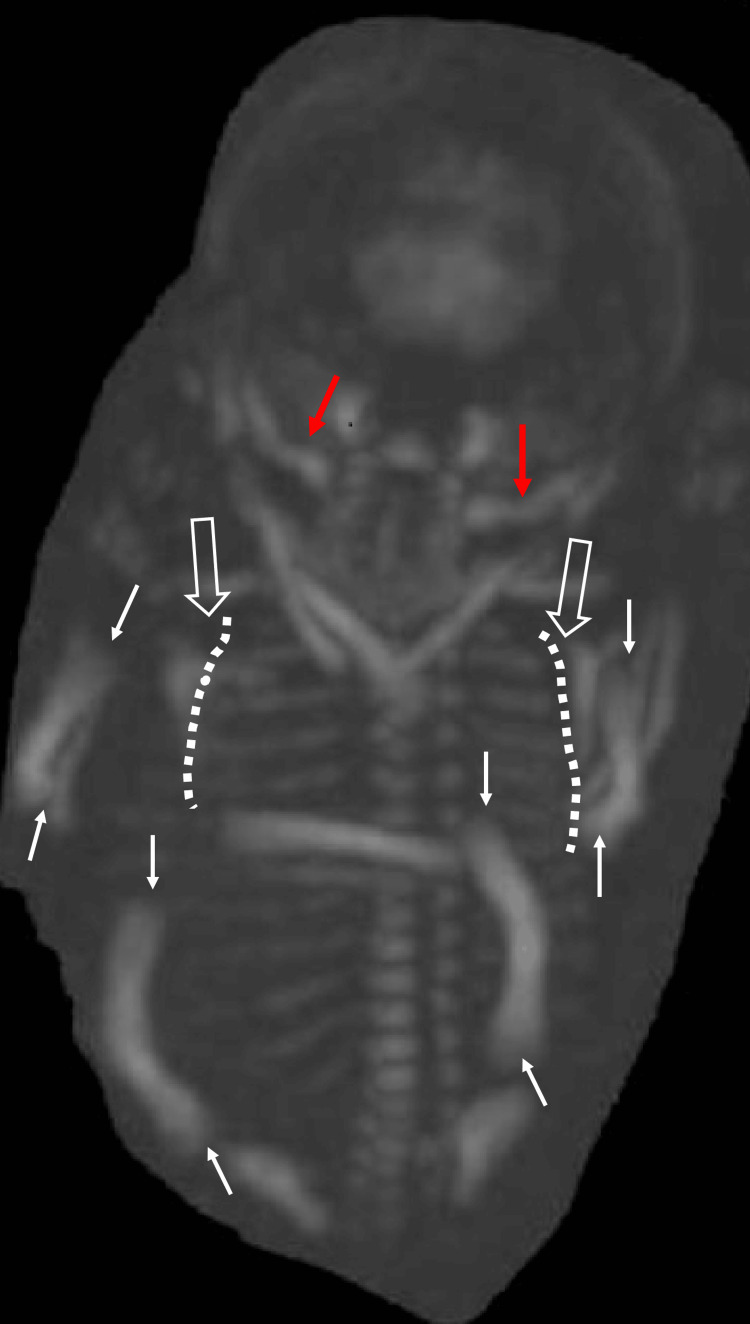
3D CT of the second pregnancy at 20 weeks and four days of gestation Fetal epiphyseal protrusion was cupping (white arrow), and the scapula was poorly detected (white open arrow), but thoracic hypoplasia (white dotted line) and periorbital ossification (red arrow) were noted. 3D CT: three-dimensional computed tomography

During her second pregnancy, at 20 weeks of gestation, prenatal ultrasonography revealed shortening and curvature of the fetal femur, humerus, and right radius and ulna (measuring approximately -2 to -3 SD below the mean), along with poor visualization of the scapula. These findings were further clarified by three-dimensional computed tomography (3D CT), which provided enhanced delineation of the skeletal abnormalities (Figure 1 and Figure 2).

Both parents had low serum ALP levels (mother: 28.7 U/L; father: 46.2 U/L; normal range: 38-113 U/L). All laboratory values and reference ranges in Table 1 are based on adult serum ALP reference intervals (38-113 U/L) as cited in the case description (Table 1). Based on these findings, and following consultation with the Japanese Skeletal Dysplasia Forum, a diagnosis of prenatal benign HPP was suspected.

**Table 1:** Longitudinal ALP measurements in the family members The ALP values measured over time are recorded. The serum ALP levels of the mother and father, as well as the fetal serum ALP levels measured at each time point during the second and third pregnancies, are recorded. Reference range for ALP: 38-113 U/L ALP: alkaline phosphatase

Laboratory test	Result (U/L)	Time point
Maternal ALP	28.7	During the second pregnancy
Paternal ALP	46.2	During the second pregnancy
Fetal cord blood ALP (second pregnancy)	40.92	At termination (second pregnancy)
Infant ALP at birth (third pregnancy)	24	At birth (third pregnancy)
Infant ALP day 11	67	Day 11 (third pregnancy)
Infant ALP 1 month	76	1 month (third pregnancy)

**Figure 3:**
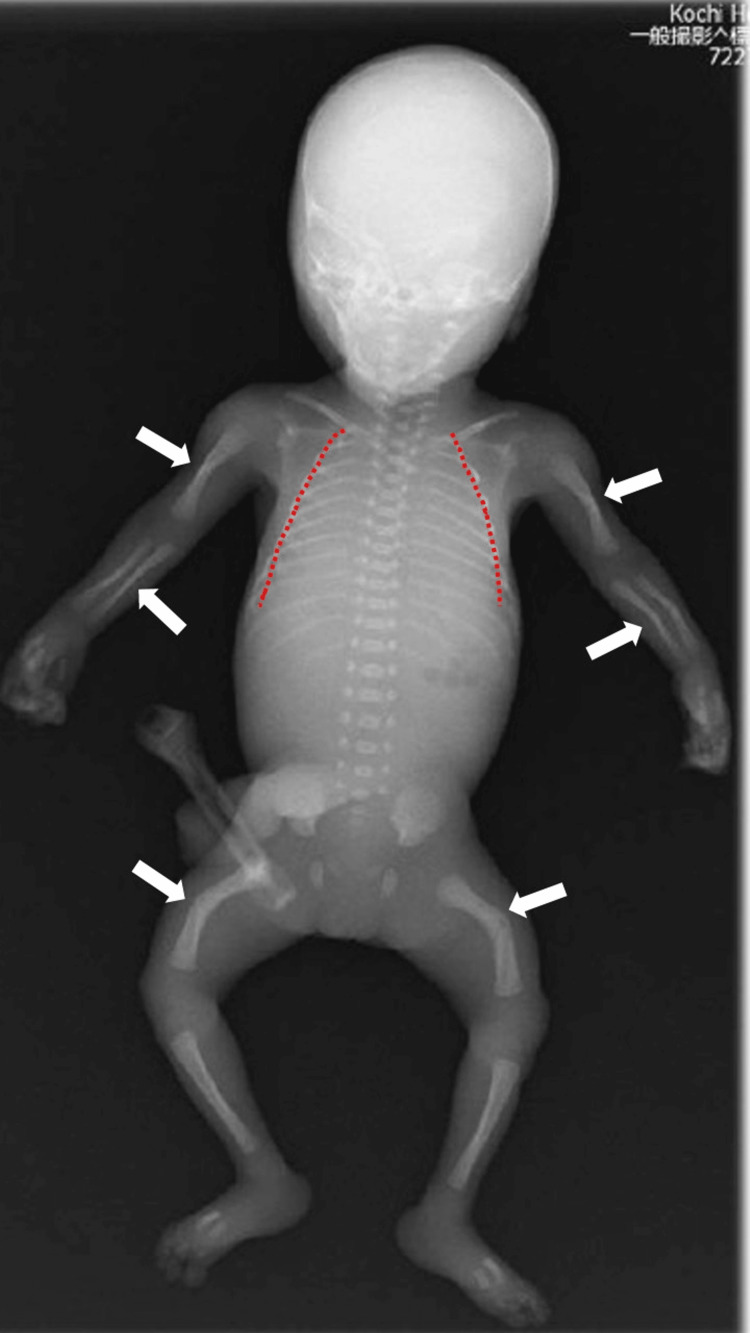
X-ray of the neonate's entire body Humerus, forearm, and femur kyphosis were noted (white arrow), with a small thorax (red dotted line).

Despite this tentative diagnosis, the pediatricians at our institution had no prior experience managing HPP cases. The initial counseling session at 21 weeks was thus provided with limited information, mostly emphasizing the negative aspects of the disease. The pediatrician stated the following: "We have no experience in treating children with HPP and are unfamiliar with the prognosis. The child may experience chronic pain, and few facilities are equipped to manage such cases." Given this, we referred the patient to a specialized facility for additional counseling via a web-based session. There, more balanced information was provided, including the possibility of a normal life without pain in cases of prenatal benign HPP. Despite this, the patient and her family opted to terminate the pregnancy at 21 weeks and five days. Post-termination investigations included a whole-body X-ray of the fetus (Figure 3), which confirmed findings consistent with prenatal benign HPP.

Umbilical cord blood ALP was 40.92 U/L, suggesting partial enzyme activity. Genomic DNA was extracted, and all coding exons of the ALPL gene were individually amplified by PCR and analyzed by direct Sanger sequencing. The patient was found to be a compound heterozygote for two mutations in the ALPL gene, confirming the diagnosis of HPP. A heterozygous c.979T>C (p.Phe327Leu) missense mutation was identified in exon 9, resulting in the substitution of phenylalanine with leucine at amino acid position 327. Additionally, a heterozygous c.1101_1103delCTC (p.Ser368del) in-frame deletion was detected in exon 10, leading to the loss of a serine residue at codon 368. According to ClinVar and the Human Gene Mutation Database (HGMD), both the p.Phe327Leu and p.Ser368del variants in the ALPL gene are classified as pathogenic or likely pathogenic (accessed June 17, 2025). The p.Phe327Leu (p.F327L) mutation in exon 9 is the second most common ALPL variant reported in Japanese patients with HPP. This mutation retains relatively high residual ALP activity (approximately 70% of wild type) and is associated with clinically milder forms of HPP, such as the perinatal benign type. In this subtype, bowing of the long bones can be detected prenatally, but skeletal hypomineralization is mild, and patients can survive without enzyme replacement therapy. The p.Ser368del (p.S368del) mutation in exon 10 is a previously reported pathogenic variant observed in cases of perinatal-onset HPP [5].

In the third pregnancy, genetic counseling was initiated early at nine weeks of gestation. Although genetic testing of the parents was not performed, a definitive recurrence risk could not be established. However, based on the clinical scenario, we explained that if both asymptomatic parents are heterozygous carriers and the affected fetus is compound heterozygous, the recurrence risk would be 25%, meaning there is a possibility that another affected fetus could inherit both mutations. After being informed of this risk, along with available diagnostic options such as amniocentesis and the spectrum of clinical severity, the patient elected to forgo invasive testing and opted to continue the pregnancy with close monitoring.

**Figure 4:**
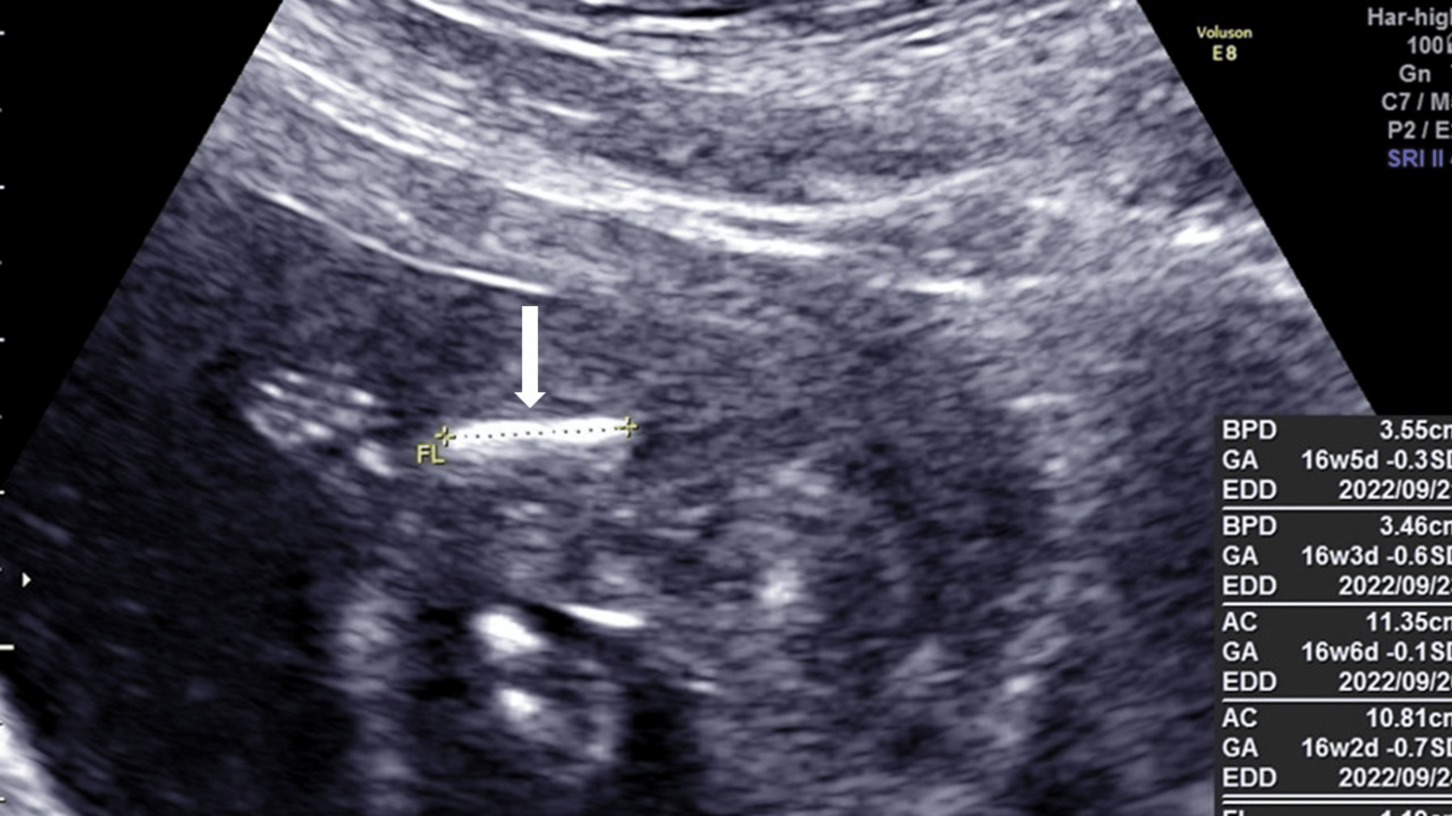
Femur length on ultrasonography of the third pregnancy at 17 weeks of gestation The fetal femur was shortened and curved (white arrow), similar to the second pregnancy.

**Figure 5:**
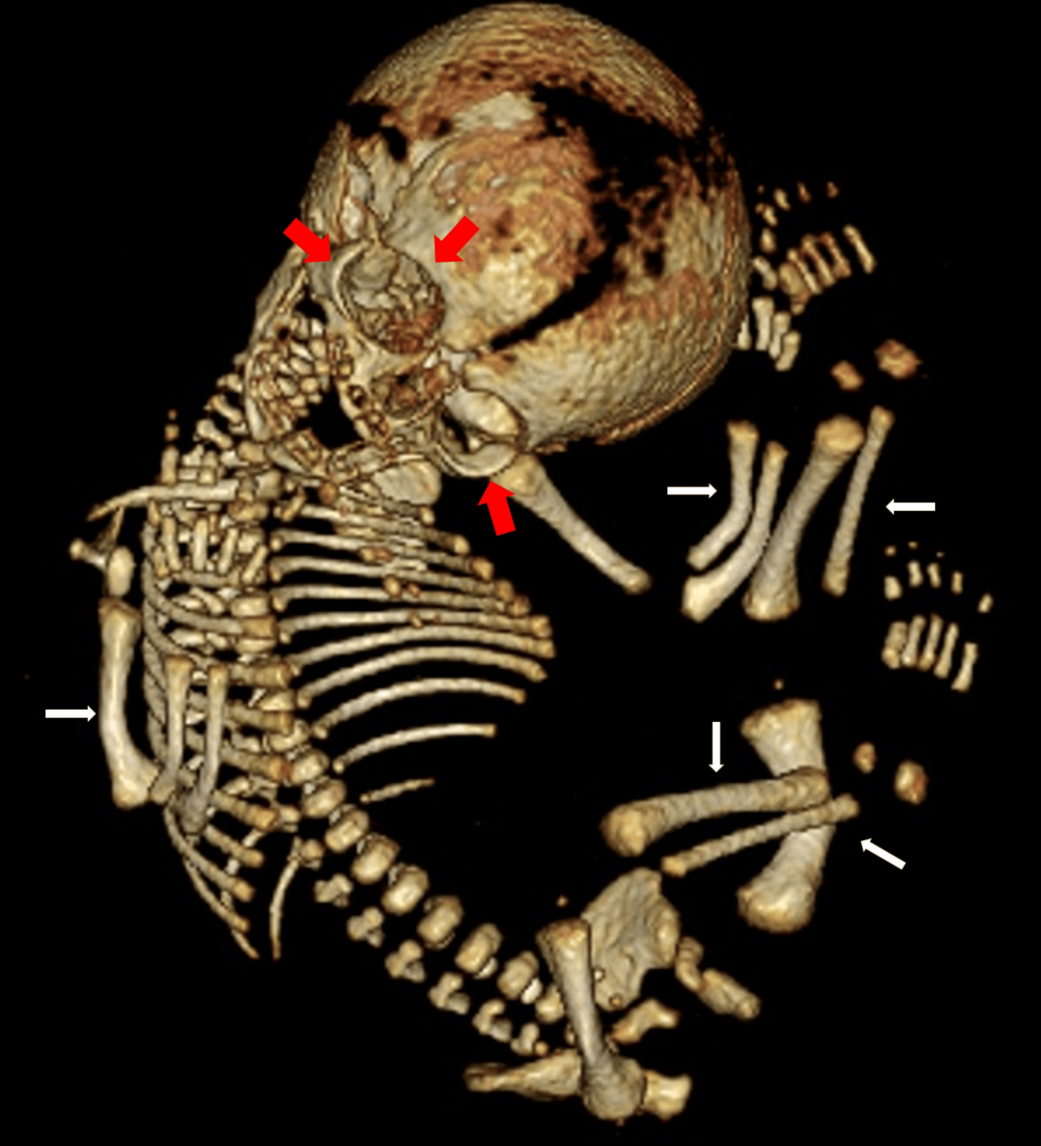
3D CT of the third pregnancy at 35 weeks of gestation The fetal long tubular bones were curved and shortened (white arrow), with mild hypoplasia of the thorax, but the skull base and orbit were not hypoplastic (red arrow). 3D CT: three-dimensional computed tomography

At 16 weeks of gestation, ultrasonography again revealed femoral shortening (Figure 4), similar to the previous pregnancy. No polyhydramnios was observed throughout the course of the pregnancy. At 35 weeks, 3D CT imaging confirmed curvature and shortening of the long bones, with mild thoracic hypoplasia but no skull base abnormalities (Figure 5). The findings were consistent with prenatal benign HPP. The pediatric team at a nearby general hospital was prepared to manage the newborn.

**Figure 6:**
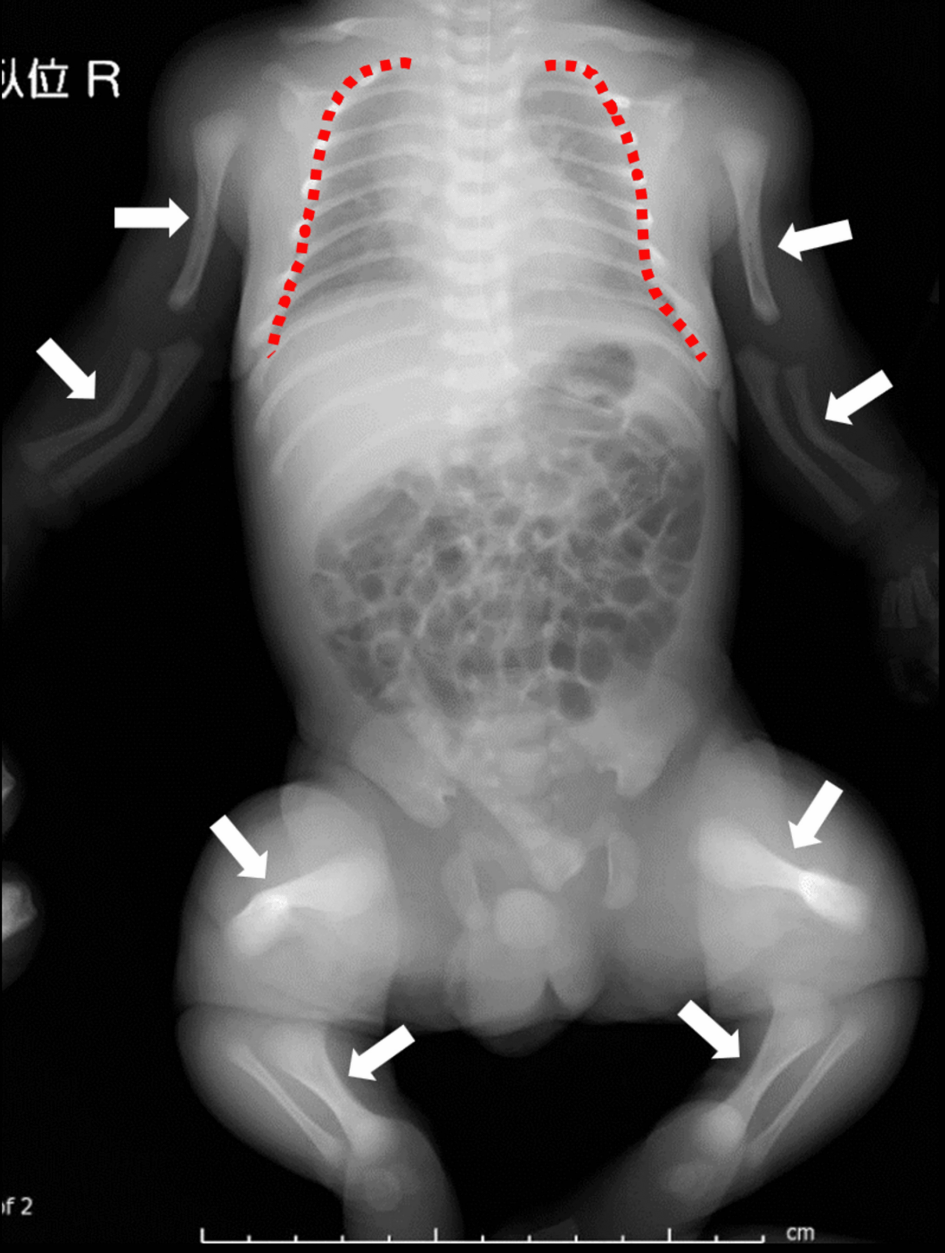
X-ray of the neonate's trunk Humerus, forearm, femur, and lower leg bone kyphosis were noted (white arrow) with a small thorax (red dotted line).

**Figure 7:**
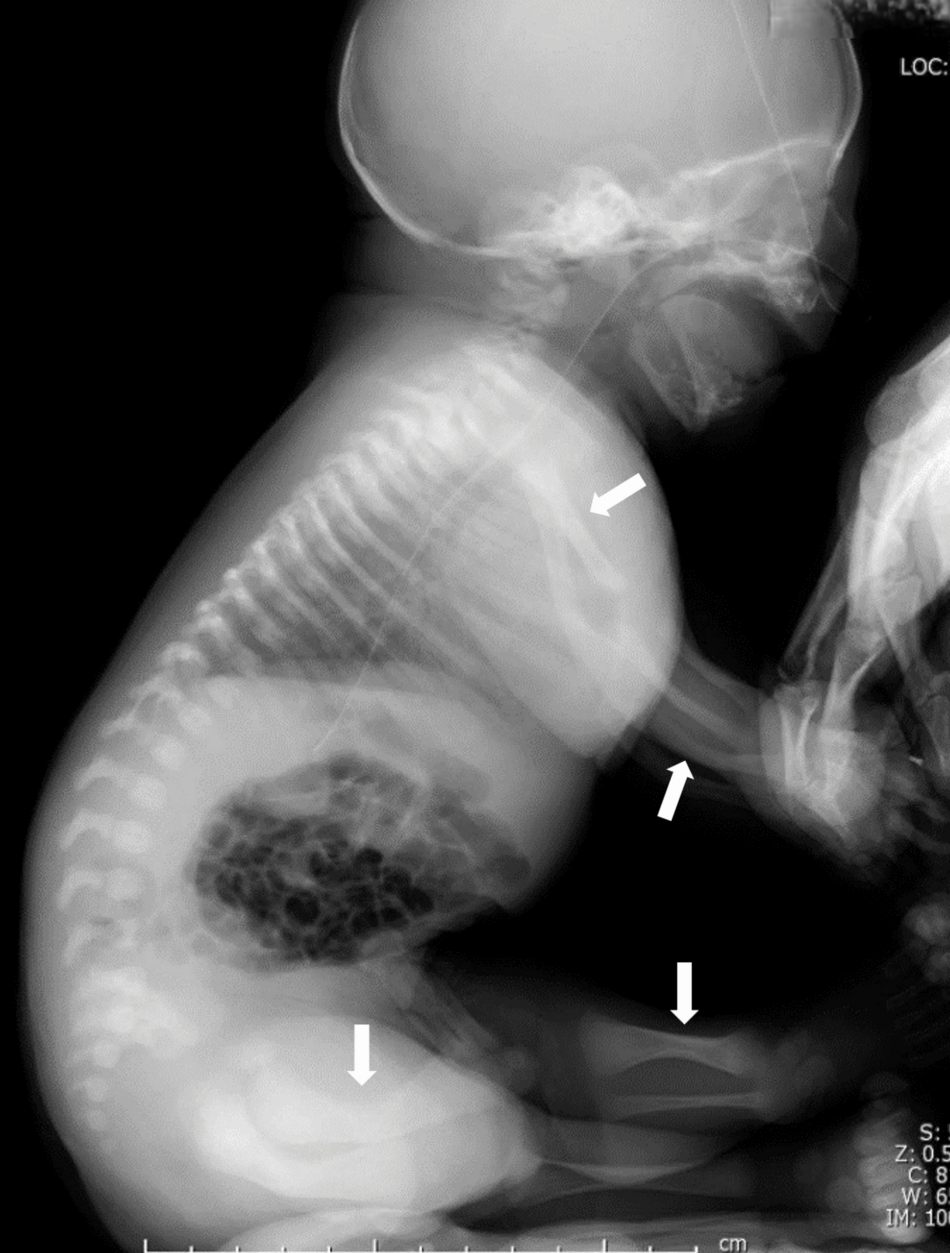
Side view of the neonate's trunk on X-ray Along with the upper arm and forearm, the long bones of the lower leg are also hypoplastic and curved (white arrow). No notable findings were observed in the vertebrae.

At 38 weeks and four days of gestation, the patient delivered a male infant via elective cesarean section due to a history of prior cesarean delivery. The neonate weighed 2608 g, measured 43.9 cm in length, and had Apgar scores of 7 and 8 at one and five minutes, respectively. While limb shortening was noted, there were no major external deformities (Figure 6 and Figure 7).

Respiratory support was required only briefly: continuous positive airway pressure (CPAP) was discontinued on day 1, and oxygen therapy was stopped on day 2. ALP levels were 22 U/L in cord blood and 24 U/L in the infant, increasing to 67 U/L by day 11. The child was discharged on day 13.

At the one-month follow-up, the infant showed normal weight gain (+70 g/day) and stable calcium and ALP levels (10 mg/dL and 76 U/L, respectively). Genetic testing revealed the same p.Phe327Leu mutation. Given the favorable course, enzyme replacement therapy was deemed unnecessary.

## Discussion

In general, prenatal benign HPP is associated with a favorable prognosis [3]. When fetal anomalies are detected, obstetricians are responsible for informing parents about the nature, severity, and expected outcomes of the condition. If the anomaly is life-threatening or severely disabling, termination may be considered depending on gestational age and local regulations. However, when the prognosis is favorable or effective treatments exist, physicians may be more inclined to recommend continuation of the pregnancy.

Recent advances in prenatal diagnostics and ultrasonography have enabled the earlier detection of congenital anomalies. Consequently, counseling regarding fetal conditions has become a routine and essential component of obstetric care. Studies have shown that decisions to terminate are most commonly associated with poor life expectancy or central nervous system involvement [8,9].

Although a direct comparison is difficult due to timing differences, our patient experienced two pregnancies with remarkably similar clinical features and prognoses. Despite this, the choices made by the patient and her family were opposite: termination in one case and continuation in the other. This highlights the critical impact of both the timing and content of counseling on decision-making.

In the second pregnancy, the initial counseling was provided by a physician lacking experience with HPP. The explanation emphasized negative aspects such as incurability and potential pain, which likely contributed to misperceptions about the condition. More balanced and positive information was only provided later through an online consultation with experts, including pediatricians who had direct clinical experience with the delivery and postnatal care of infants with perinatal benign HPP. However, due to the limited time window before the legal abortion limit (22 weeks in Japan), the family was unable to fully process the updated information and reconsider their decision.

In contrast, during the third pregnancy, counseling began in the first trimester, and the patient was given adequate time and opportunity to consider all options. A multidisciplinary team, including experienced pediatricians, genetic counselors, and local follow-up providers, supported the family throughout the pregnancy. The cumulative effect of early, balanced, and supportive counseling allowed the patient to make an informed decision to continue the pregnancy.

Two major factors influenced the outcomes in this case: the accuracy of the information provided and the timing of its delivery. Counseling should present a balanced view, including both potential challenges and favorable outcomes. Information is most effective when delivered by professionals with direct clinical experience or, if unavailable, by providers committed to helping families navigate decisions with empathy and responsibility.

Timeliness is equally critical. Parents require time to process complex information, ask questions, and reflect on their values and priorities. The frequency and duration of counseling, as well as early initiation, may be more important than a single in-depth session. Team-based support involving midwives and psychologists can further enhance decision-making and psychological well-being.

Although HPP is rare, especially in its prenatal benign form, it is not considered a life-threatening or severely disabling condition. Most children with this subtype can live normal lives with appropriate monitoring. In the case presented, early and accurate counseling made the difference between pregnancy termination and successful delivery (Table 2).

**Table 2:** Comparison of counseling characteristics between Case 1 (second child) and Case 2 (third child) The table summarizes the differences in counseling approaches and parental responses between Case 1 (second child) and Case 2 (third child), both prenatally suspected of HPP. In Case 1, the first visit occurred at 20 weeks of gestation, and counseling was provided at 21 weeks, shortly before the legal limit for pregnancy termination in Japan (22 weeks). The absence of specialized genetic input led to the delivery of incorrect and overly pessimistic information, which caused significant anxiety and confusion during a time-constrained decision-making process. In contrast, Case 2 involved an initial visit at nine weeks and counseling at 16 weeks, allowing sufficient time for multidisciplinary evaluation and comprehensive information sharing, including the possibility of a benign form of HPP. This led to better parental understanding and psychological preparedness. The comparison underscores the importance of early referral, genetic consultation, and accurate, multidisciplinary counseling in pregnancies involving rare genetic conditions. HPP: hypophosphatasia

Item	Case 1 (second child)	Case 2 (third child)
Timing of first visit	20 weeks of gestation	9 weeks of gestation
Timing of counseling	21 weeks of gestation	16 weeks of gestation
Counseling providers	Obstetrician and pediatrician (without specialized knowledge in medical genetics)	Obstetrician, pediatrician, and a multidisciplinary team including medical geneticists
Quality of information provided	Information was limited to the general possibilities of fetal skeletal dysplasia. Incorrect and overly pessimistic information was included, without any mention of the benign form of HPP	Detailed explanation of HPP, including its clinical spectrum, inheritance pattern, possibility of the benign perinatal form, and perinatal management strategies
Timing of information delivery	Counseling was conducted shortly before 22 weeks of gestation, the legal limit for pregnancy termination in Japan, which placed the family under significant time pressure	Information was provided well in advance of the legal decision-making deadline, allowing time for reflection and understanding
Parental understanding	Inadequate understanding of the condition; strong anxiety. The pessimistic counseling and legal time constraint led to confusion and emotional distress during decision-making	Relatively good understanding of the disease and its prognosis, enabling calm and informed decision-making
Support system	Limited to the involvement of an obstetrician and a pediatrician	Well-coordinated support involving obstetrics, pediatrics, genetics, and the perinatal care team

This case emphasizes the need for structured, multidisciplinary support systems in prenatal care, especially for rare diseases. Personalized, timely counseling can empower families to make choices aligned with their values, even in the face of uncertainty.

## Conclusions

This case highlights the critical importance of the timing, content, and delivery of prenatal counseling in pregnancies complicated by rare conditions such as HPP. Even when the same disease is diagnosed with similar clinical findings, the decision to terminate or continue a pregnancy can differ significantly depending on the quality of information provided and the support system in place.

To support optimal decision-making, counseling should begin early and be based on accurate information delivered by a multidisciplinary team. This approach supports the autonomy of pregnant women and their families and ensures decisions are based on comprehensive and balanced information. In rare diseases like HPP, the presence of a structured support system can greatly improve outcomes by enabling informed and confident parental choices.
